# Understanding the drivers of poor infection prevention control (IPC) practices in Kenyan health facilities: An interdisciplinary study

**DOI:** 10.1371/journal.pgph.0004404

**Published:** 2025-06-12

**Authors:** Hannah Brown, Aloyce Odhiambo, Alex Mwaki, Nancy Atieno, Rosebel Ouda, Isaac Ngere

**Affiliations:** 1 Anthropology Department, Durham University, Dawson Building, South Road, Durham, United Kingdom; 2 Safe Water and Aids Project (SWAP). Off Aga Khan Road, Behind Royal City Garden Hotel, Milimani Estate, Kisumu, Kenya; 3 Paul G. Allen School of Global Health, Washington State University, Pullman, Washington, United States of America; 4 Washington State University Global Health Program, Washington State University, Nairobi, Kenya; University of New South Wales, AUSTRALIA

## Abstract

Improving IPC practices in health facilities is a major concern for the global health community. This paper combines insights from rapid ethnographic research and epidemiological measures of IPC adherence undertaken in four health facilities of different sizes in western Kenya between November 2022 and January 2023. At the time of our research, the Covid-19 pandemic was a WHO-defined Public Health Emergency of International Concern, although the pandemic was subsiding, and there was an ongoing Ebola outbreak across the border in Uganda. Our findings evidence serious issues with adherence to standard IPC guidelines and the urgent need for reform of IPC practices. If the practices we observed are widespread beyond the study setting, we believe there is an urgent need to develop new interventions to improve adherence to IPC guidelines, with better follow-up to ensure interventions have long-term impact. The ethnographic material we present offers insights into the following IPC-related practices: (1) Hand hygiene (HH) and glove use; (2) Use of objects/material items; (3) Cleaning; (4) Waste disposal. When interrogated alongside epidemiological findings, our data offer preliminary evidence for understanding some of the social drivers for poor IPC adherence and the ways risky practice is embedded in organisational processes and personal perceptions of risk. We highlight the need to consider the social context of IPC behaviours in order to develop interventions which can deliver sustained change. Specific recommendations include better training and resources for cleaning and casual staff, and for a shift in emphasis in IPC interventions from health worker education towards modes of influencing sustained behaviour change.

## Introduction

The past decade has seen significant investment in Infection Prevention Control (IPC) activities across sub-Saharan Africa, including the development of global and national guidelines; increase in availability of basic equipment; and improvements in Water, Sanitation and Hygiene (WASH) infrastructure. More recently, the COVID-19 pandemic led to significant changes in WASH and IPC policy and widespread new investments [1,2]. However, major challenges remain. For example, in Western Kenya, where this study was carried out, facility assessments have shown that despite improvements in WASH and IPC infrastructure and acceptable levels of knowledge among staff about *how* to use IPC, *when* to practice IPC, and *why* IPC is important, adherence to hand hygiene standards among healthcare workers was still lower than 20% [3]. Additionally, whilst recent investments in IPC are to be welcomed, the longevity of improvements in IPC infrastructure and policy initiated during COVID-19 is unclear. Literature suggests that inadequate access to basic IPC resources and the proper use of available materials are both significant issues, and that there is frequently a large gap between the adoption of new policy and seeing improvements on the ground. For example, according to the 2021–22 Tripartite Antimicrobial Resistance Country Self-assessment Survey (TrACSS), Kenya is among Africa countries that have made the most progress in the development of a national IPC programme [4]. However, the Kenyan Ministry of Health simultaneously describes a situation of “alarming gaps…in the provision of basic hygiene and handwashing services in health care facilities across many countries.” [5] Meanwhile, in Kenya and elsewhere, shortages and stock-outs of essential equipment and basic supplies remain common, forcing health workers to develop workarounds to deliver the best care they can in seriously deficient circumstances.

The implications of these gaps in IPC are significant for health worker, patient, and community wellbeing. They raise a number health-related concerns, particularly in terms of epidemic control, the likelihood of Healthcare Acquired Infections (HAI), and a concurrent growth of Antimicrobial Resistance (AMR) due to the need for treating avoidable additional infections [4,6]. Countries in the global South are particularly at risk. In Low and Middle Income Countries, 15% of patients in acute care hospitals will develop one or more HAI during hospitalisation, many of which are linked to poor hygiene as a result of lack of effective IPC, and globally, sepsis causes around 11 million deaths each year [7]. Improving IPC is therefore a major priority for the global health community [4,8]. Health workers and other hospital personnel are currently putting themselves, their families, their patients and the wider communities at huge unnecessary risk. In a global environment where pandemic disease is becoming more common, many health facilities also appear to be grossly unprepared to deal with future emerging diseases.

In this paper, we draw on anthropological and epidemiological approaches to make the case that better understanding of the drivers and social context of poor IPC behaviours is crucial for developing interventions that can improve the uptake of recommended practice and guidelines. We know from previous ethnographic studies that healthcare guidelines often fall short in their capacity to ‘guide’ health worker behaviour because they fail to account for social realities. For example, when gloves are in short supply, is it better to carry out a procedure with one glove, or to save gloves for more ‘risky’ procedures? If there is no bleach, is it better to wash a stethoscope in ‘plain’ [i.e., untreated] water, or not clean it at all [9]? We also know that health workers often follow ‘mindlines’ as much as guidelines, interpreting formal guidelines in the light of local values, relationships and resource scarcity that shape “collectively reinforced, internalised tacit guidelines”[10]. Ethnographic research has also shown that the use of gloves and PPE is often shaped as much by the context and relationship in which these items are deployed as it is by knowledge about how to use them [11]. This paper builds on this existing literature and further develops our understanding about *why* people adopt behaviours that may not align with WHO guidelines. Importantly, we argue that better understanding of social drivers of IPC practice and the contexts that shape them provide a range of important insights for future interventions that can improve IPC cultures in Kenya and beyond.

## Methodology and methods

### Study site and setting

We carried out the study in four health facilities in Kisumu County, which were purposively selected as representative of different sized facilities that constitute the Kenyan government health system (one dispensary, a health centre, a sub-county hospital and a county hospital)[12]. The sub-county hospital and the county hospital have inpatient facilities and are the referral facilities for the lower facilities. The four facilities are located two sub counties in rural Kisumu County and were all implementing the Universal Healthcare Package where basic health services were offered for free. A US Centres for Disease Prevention and Control (CDC) and Washington State University funded hand hygiene project involving provision of hand hygiene stations, supplies such as alcohol based hand rub (ABHR), liquid soap, and follow-up observations had been implemented in these facilities since 2021 and was recently completed at the time of fieldwork [3].

### Study design

Challenges with IPC in resource-limited settings like Kenya are a complex problem, as much socio-cultural as they are structural, political and economic. Such problems exceed the capabilities of any single discipline and demand an interdisciplinary approach to the ways that behaviours, material cultures, supply chains and infrastructures intersect [13]. The combination of ethnographic and epidemiological insights developed in this paper are designed not only to measure IPC adherence and non-adherence, but to go ‘beyond checklists’ and understand how IPC practices unfold in the context of local working environments [14]. This study used a mix of ethnographic approaches with epidemiological assessments of IPC behaviours. We observed for the availability and usage of hand hygiene supplies at entrances and exits and patient care rooms in the four HCFs, observed the performance of HH by selected staff and assessed for hand cleanliness by healthcare workers to determine the effectiveness of the IPC and HH practices. We also spent time observing healthcare workers in their everyday work and talking to them about IPC and HH.

### Ethnographic data collection

The ethnographic fieldwork component was carried out by a team of 3 researchers and led by HB, an anthropologist with long-term expertise carrying out research in the Kenyan health sector. The other members of the ethnographic team were researchers who were experienced in a range of qualitative methods but no previous experience of participant observation, the primary ethnographic method. Prior to the fieldwork HB led a one-day training course on ethnographic research methods that was held at the Safe Water and AIDS Project (SWAP), a research-active Non-Governmental Organisation (NGO) based in Kisumu, Kenya. HB accompanied the researchers on initial field visits, reviewed fieldnotes, and discussed experiences of using ethnographic methods and initial findings, until confident that the researchers were able to work independently. We spent two days in each of the four facilities. The ethnographic team held daily debriefs following fieldwork to discuss emergent findings. We made preliminary handwritten notes in the field, which were typed up in MSWord soon after our fieldwork. We also took photographs. In addition to handwritten notes, we developed a shared online note-taking system using the Trello application.

### Epidemiological data collection

Alongside the ethnographic fieldwork, we collected epidemiological survey data and took measures of observed IPC practices by healthcare workers during patient care processes, and availability and use of WASH and IPC infrastructure in each facility and patient care room against national and WHO standards [1,2]. Patient care room was defined as a room in a HCF where HCWs either had physical contact with patients, handled patient specimens or medication, donned or doffed personal protective equipment (PPE) or where patients checked-in to stay overnight. Epidemiological data was collected by two trained research assistants (RAs) through face-to-face interviews with health facility in-charges, or their nominees if the facility head was not available. An electronic questionnaire running on ODK KOBO collect platform and mounted on android OS tablets was used in the data collection. The survey tool collected data on facility details including name, level of care, location, number of visitors, number of entrances, exits, latrines, patient care rooms, IPC supplies including gloves, masks, water sources and availability of IPC and HH policy. The researchers also made observations of availability of IPC and HH supplies in patient care rooms that were accessible on the day of the survey, at entrances/exits and near latrines. During the observations, researchers recorded the HH materials available in each patient care area/room, e.g., HWS, soap and running water or ABHR in a working dispenser against a checklist developed for the study.

### Assessment of hand cleanliness

**Fig 1:**
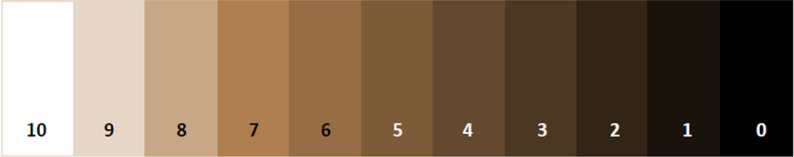
Quantitative Personal Hygiene Assessment Tool (qPHAT): An Eleven Point Color Scale.

On the day of the health facility survey, we also evaluated health care worker, patients and visitors’ hand cleanliness using simple point-of-care tools that have been validated and calibrated as methods that provide an objective measure of the effectiveness of IPC [15,16]. Briefly, after obtaining participant consent and ensuring the participant was an adult (aged ≥18 years), the participant was interviewed to assess demographics and hand hygiene knowledge. After completing the questionnaire, another enumerator collected the hand swab from the participant’s right hand. HCWs were identified for participation using two different methods depending on the size of the facility. If a facility had six or fewer HCWs (dispensaries and HCs), all of them were enrolled. For larger hospitals, a multi-stage random selection method was used to select departments within the HCF and then up to seven individual HCWs in that department who were present on the day of the visit. Interviews and hand swabs of HCWs were collected prior to patient care or once HCWs were no longer with a patient. We also randomly sampled adult patients for hand swabbing from various service points including outpatient and inpatient areas, corresponding to areas where HCWs were sampled. For visitors, we intercepted, consented and sampled adults accompanying patients to hospital on the day of sampling. To quantify hand dirtiness, we used the Quantitative Personal Hygiene Assessment Tool (qPHAT), which involves collecting a hand swab by tracing a Hygea (PDI, Orangeburg, New York, USA) saline wipe in a standardized pattern over the palms and fingertips of one hand. Hand swabs were scored by two onsite enumerators by comparing the darkest half-inch square to an 11-point color scale (Fig 1) ranging from zero (most visibly dirty) to ten (no visible dirt). To score, swabs were placed on a flat surface next to the qPHAT scale. If a swab appeared to fall between two values (e.g., between 3 and 4) then the enumerator recorded the smaller number. Any relevant observations and comments were recorded for later assessment as needed. Photos were captured of the swab and qPHAT scale using phone cameras of ≥12 megapixel and reviewed by a third reviewer for quality and clarity within three days of sample collection. The third reviewer also spot-checked the entry scores to the photos to ensure the enumerators were rating the swabs properly. Any concerns for scoring or photo clarity were brought back to the enumerator to incorporate into the fieldwork.

### Data analysis

We hypothesised that combining ethnographic and epidemiological methods would not only allow us to compare measures of IPC practice with observations and insights into the contexts shaping particular behaviours, but would also offer insight into changes in behaviour that related to our research presence, allowing us to mitigate for such effects in our analysis.

For the ethnographic data on Trello, we colour coded photographic and other observations against field-site locations and a preliminary set of thematic codes. These preliminary themes were developed by HB to help fieldworkers pay attention to the often mundane, everyday practices that shape IPC in health settings and were aligned to the ethnographic tools that we developed for fieldwork that were submitted with our ethics applications. Our preliminary themes were designed based on previous experience doing hospital-based ethnographic fieldwork (e.g., see [17]) and awareness of WASH and IPC practices in health facilities. Our preliminary thematic codes were: Cleaning, hand hygiene, touch, environment, use of material objects, PPE, and waste disposal. Once all the data was collected, HB carried out a manual thematic analysis of all the written fieldnotes and a re-analysis of the observations recorded on Trello. This reanalysis process allowed us to exclude overlapping themes and to see which themes emerged most prominently in our analysis. In this process we reduced the original themes to the following core themes which appeared with the highest frequency: (1) Hand hygiene; (2) PPE use and supplies; (3) Cleaning; (4) Waste disposal.

In order to characterize needed HH materials, epidemiological data was collected on existing HH material availability and percentages or proportions computed for the various categories or patient cares rooms and compared between healthcare facilities. For the hand swabs study, we assessed the major outcomes (knowledge of HH and qPHAT score) by the various variables including age, gender, cadre, department/type of room and presented the descriptive statistics for each category. Median scores and interquartile ranges were computed for various participant categories (healthcare workers vs patients vs visitors). The qPHAT method has shown an excellent inter-rate reliability across all types of rater comparisons (PMID: 32278303), hence inter-rater reliability was not assessed.

### Research ethics

The assessment was approved by Durham University Anthropology Research Ethics Committee (Ref *ANTH-2022-10-28T15_33_18-qfvj43)* the Maseno University Ethics Review Committee (Approval MUERC/00717/21). We received a National Commission for Science Technology and Innovation (NACOSTI) research license ref # 760161 and license # NACOSTI/P/23/22837. Administrative approvals were also obtained from the Kisumu County health department. The study took place between 6^th^ December 2022 and 13^th^ January 2023. Verbal consent was obtained from all participants prior to questionnaire interview and ethnographic engagement. Consent was recorded in field notes and re-obtained on each new visit to a health facility.

## Results

### Description of the health facilities, IPC and WASH access

As shown in Table 1, four health facilities that were visited had an average of 1,500 visitors, 99 in patient admission and 68 deliveries every month. The majority of staff in the four facilities were clinical staff (nurses, clinical officers, doctors and laboratory staff) when compared to non-clinical staff (cleaners, administrators, security staff, clerks etc). The main source of water in 2/4 (50%) of the health facilities was a borehole with a hand pump and in all the facilities, water source was located more than 500m from the compound. Two HCFs had hand hygiene policies in place whose compliance was charged to healthcare workers.

**Table 1:** Characteristics of the 4 healthcare facilities in Kisumu county, 2022.

Variable	Number (%) N = 4
**Facility workload (monthly averages)**	
Monthly outpatient numbers	1,506
Monthly admissions	99
Monthly deliveries	68
Visitors	175
**Staffing**	
Clinical staff	33
Non-clinical staff	20.5
**Main water sources for handwashing (per facility)**	
Piped water (tap or standpipe)	1 (25.0%)
Borehole with hand pump (has a small diameter and was dug with a machine)	2 (50.0%)
Rainwater harvest tank	1 (25.0%)
**Where water is accessed - within compound or outside**	
More than 500 meters from the health facility	4 (100.0%)
**Interruptions to water supply**	
No	4 (100.0%)
**Availability of hand hygiene policy**	
Yes	2 (50.0%)
No	2 (50.0%)
**Who ensures there is compliance to HH policy?**	
Healthcare workers	2 (50.0%)
Receptionist/cleaner	2 (50.0%)

### Hand Hygiene

**Fig 2:**
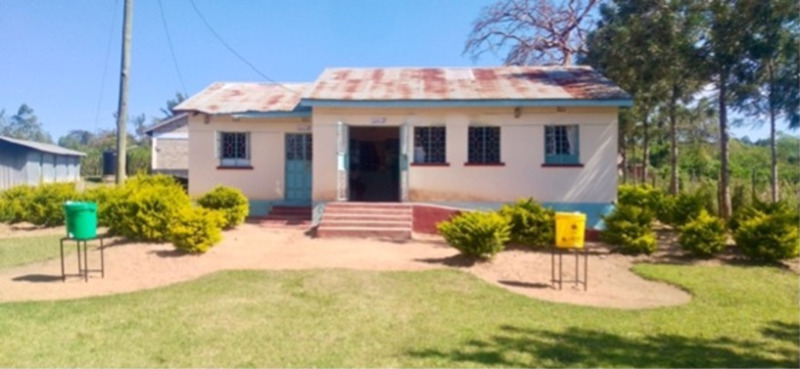
Veronica buckets for handwashing outside a health dispensary. Liquid soap is available in plastic bottles on top of the large buckets (Photo credit: **H.**Brown).

**Fig 3:**
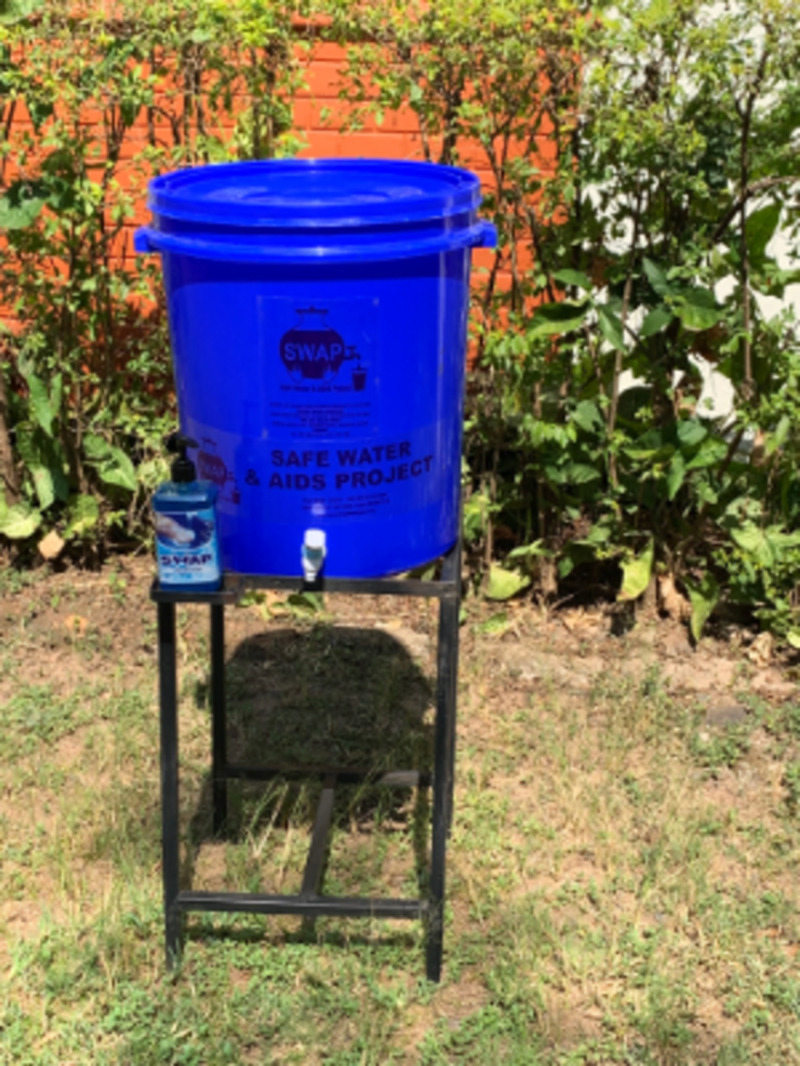
Typical example of Veronica Bucket on a stand of the kind used in the health facilities (Photo credit: SWAP).

All the four HCFs assessed had handwashing stations, which were mainly modified veronica bucket types at entrances, near exits and latrines. During the Covid-19 pandemic, health facilities in our study had been supplied with veronica buckets for use at handwashing stations and soap (see Figs 2 and 3). We found that these items were still in place at the facilities we visited, and were mostly placed strategically for example near entrances to the facility, latrines, and wards, although in some cases there were no handwashing stations near latrines. The handwashing stations that we tested all contained water, with health worker (50%), casual staff (25%) and cleaners (25%) responsible for cleaning the stations and keeping buckets full. HWS were reportedly cleaned either daily (50%) or weekly (50%) depending on usage.

In most cases soap was available on the handwashing stations, but not consistently. At one health facility we checked the handwashing stations when we arrived and found that each one had a brand new, unopened container of liquid soap (we assumed these had been newly placed on the buckets in anticipation of our visit). At one of the larger health centres, staff complained that liquid soap sometimes went missing. They had improvised ways of attaching soap bottles to the handwashing stands using string. There were no hand hygiene facilities available inside any of the patient wards that we visited, rather, handwashing buckets were placed outside of buildings at the entrance to the wards, usually in a place where the grey water could run onto the ground. This meant that health staff usually did not practice HH in between examining different patients as no hand hygiene materials were easily available. Staff in all the facilities had received training on handwashing station management, provided by SWAP as part of an ongoing WASH project.

Wall mounted ABHR did not exist in any of the health facilities that we visited, and we did not observe any staff carrying small bottles of personal ABHR to use during their work. All the facilities we visited had sufficient stocks of ABHR in place during our visits, mainly supplied by SWAP (75%). Sometimes this was available on desks close to patient care areas, but on other occasions ABHR was awkwardly placed on the tops of cupboards or out of reach, and in many instances, although ABHR was available it was not being used. Some health care workers did not use ABHR before or after patient contact, even when it was placed near them and easily within reach. ABHR stock outs were reported in three quarters of the health facilities, with delays in replenishing stock being cited as the comments reason for the stockout. In all the four facilities, ABHR was provided for use by both clinical and non-clinical staff. Management of the ABHR refill was exclusively a role of the clinical staff in all the four facilities.

We observed the entrances and exits of the four facilities for availability and usage of handwashing stations and ABHR. All entrances had handwashing stations with soap and water while none had ABHR. Most patients did not wash their hands when entering facilities or did not wash hands properly. For example, we saw one woman enter and wash only one hand, in the manner that sometimes people do when they are eating. In another case, a family entered a health facility and stood and waited whilst the father washed his hands before following him inside, “as if it’s the father who is responsible for washing hands for the whole family”, our researcher commented in her fieldnotes. In one health facility, a community health volunteer (CHV) reminded patients that they should wash their hands when they entered the facility, but her reminders were intermittent, and we suspected that she was making an effort to remind people partly because of our presence. Of the 12 latrines that were observed, only three (25%) had hand washing station with soap and water nearby. We witnessed many patients using latrines and not washing their hands afterwards, and others who washed their hands but did not use soap. There was no signage in any of the health facilities to remind people to wash hands after using latrines.

A total of 67 patient care rooms were accessed for observation. Of these, 36 rooms (53.7%) had functional and stocked ABHR dispensers while 26 rooms (38.8%) had functional handwashing stations with soap and water. Given that handwashing stations were fewer and mostly placed outside of buildings in grassy areas, hand sanitiser was a more appropriate option for use by health workers during patient consultations. However, only a few health workers used hand sanitiser consistently during patient contact. There were many instances we observed where gloves and hand sanitiser were available on consultation desks and neither was used.

“Sanitizer was on the table…but had not been opened, it was still new and the nurse did not bother to use it even after attending to patients” (Fieldnotes, 8^th^ December 2022)

“Nurse does not perform hand hygiene before or after attending to patients. Some contacts included injection…done with bare hands” (Fieldnotes, 8^th^ December 2022)

In laboratories and maternity wards, we observed health workers using gloves but sometimes only changing them after touching a number of patients. One researcher wrote in her fieldnotes, “The lab technician puts on gloves, pricks about five to six patients using the same gloves, takes the samples in for processing, removes the gloves, washes hands, and puts on new gloves for another batch of pricks.” The recommended practice would be to change gloves and perform hand hygiene between patients. Another researcher on a paediatric ward observed,

“The nurse put on gloves…to inject a child…The nurse injected three children using the same pair of gloves (she did not change the gloves). After she finished injecting the children she…removed the gloves, then she used the hand sanitizer that was on top of the trolley” (Fieldnotes, 9^th^ Jan 2023).

Hand hygiene was more regularly practiced during patient and laboratory consultations than it was at reception and triage areas. In reception areas, health care workers, often volunteers or low-paid casual staff, took the height and weight of patients and recorded this information, and sometimes carried out other tasks on behalf of qualified health workers, often touching many patients and surfaces without practising any hand hygiene.

“The receptionist is a CHV (Community Health Volunteer) and…pricks the patients [for rapid malaria tests] without practising hand hygiene, despite the fact that the sanitizer is just at the table that she operates from.” (Fieldnotes, 9^th^ January 2023).

When we asked facility staff why they did not use gloves more often, many of them told us that gloves often ran out. One researcher summarised a conversation with a nurse who explained to her,

“They do not have enough glove supply and that’s why he sometimes works without gloves. He considers wearing gloves essential for very critical cases like delivery and wound cleaning. So according to him, the pricks [for malaria testing] are minor cases” (Fieldnotes, 8^th^ December 2022)

Another made a similar comment,

“Gloves are a problem [i.e., because the supply is intermittent] and so we don’t use them most of the time. We gauge when to use and especially when interacting with patient body fluids…for example when cleaning a wound or helping a mother to deliver”. Nursing Officer in-charge of a maternity ward, 13^th^ January 2023.

In one case we were able to follow up with a nurse to discuss his hand hygiene practice. We had observed him attending to a number of patients and carrying out a range of tasks including taking blood pressure, temperature and doing malaria tests without performing any hand hygiene. Among his patients were a woman who was coughing vigorously and expectorating phlegm into a cloth. When he examined her, he touched her mouth with his bare hands. Curious about his behaviour, our researcher took the opportunity to ask him whether he was concerned about the risk of infection in these encounters. His answer combined recklessness and fatalism when he laughed and said, “It can happen”, but shrugged off the risk.

### Material culture

Our observations not only took in hand hygiene, but also closely observed the many different objects that health workers touched as they went about their work. At one health facility, HB observed a casual worker coming in and out of the consulting rooms and helping the nurses by collecting medicines and other items they needed. She also held babies so that people could stand on the scales, wrote in books for recording attendance, occasionally searched for a stamp for the health facility that seemed to be commonly required by many different people, and carried out basic bureaucratic tasks at the reception. All of this work was done without practising hand hygiene. Indeed, waiting areas and consultation rooms were often packed with different objects including weighing scales, height meters, booklets, books and pens and rubber stamps, often touched by many different people, who were also touching patients as they arrived to receive care, none of which we ever saw being disinfected. The recommended practice would be to practice hand hygiene between patients and between different contact instances, e.g., when touching inanimate objects or when moving from a contaminated to a less contaminated site in a patient’s body.

On more than one occasion we saw staff or patients eating in consulting rooms. In one health facility bananas were being sold from a desk where patients were also receiving care. On the wards, where food was sometimes provided, we observed family members supporting patients to eat and take medicines, often without washing hands,

“One patient was in a dire situation…her mother and sister had to sit her up in order to aid her take medicine. I saw them…forcing medicine into her mouth and …. the mother put her fingers in the patient’s mouth as if to try and open her teeth which she had closed tight. She did this without performing any hand hygiene.” (Fieldnotes, 15^th^ December 2022)

We also observed a number of activities relating to hospital beds. On more than one occasion, we saw casual staff rushing to make up beds with sheets and blankets when we arrived at health facilities even though no patients were admitted, suggesting to us that they had not received training on when to get beds ready for patients, and assumed that visitors would expect to see beds fully made up. In some cases, patients in paediatric and maternity wards were sharing beds, often using bedding that had been brought from home. When wards were busy, beds were not always made properly, and patients were often found lying on bare mackintosh fabric. We did not observe the laundry process for hospital bedding because members of our team had recently been involved in a separate study that looked at laundry practices in an overlapping study site.

Health staff frequently used gloves, and gloves were available in all the health facilities where we worked. We did not see other forms of PPE being frequently used. Some health workers wore masks but this seemed to be a matter of personal preference. We saw one cleaner wearing an apron for her work but another complained, “they do not have enough PPE like aprons and mask but at least gloves are always available” (*Cleaner, 13*^*th*^ *December 2022*).

### Cleaning

Cleaning was a major focus of our observations and emerged as an area of serious concern. Cleaning responsibilities were shared between healthcare workers, casual staff and volunteers. For example, in 2 (50%) of the health facilities, healthcare workers were responsible for cleaning and decontamination of handwashing stations. Cleaning of patient areas was mostly done daily or weekly. We were told that hospitals employed staff on casual contracts no longer than three months long because they could do so without having to take on these staff on permanent contracts with higher salaries. This meant that there was a high turnover of casual staff. All the casual staff we found working in the health facilities were women. Some of these women told us that they had not been trained in how to clean the health facility. Others said they had been trained in cleaning, but on further discussion it seemed that this training mostly involved being told *what* to clean rather than *how* to clean, or how to clean safely. For example, when asked how she did the cleaning at hospital, one woman told us, “*Unafanya vile unafanya nyumbani”,* i.e., “You do it the way that you do it at home”, and went on to say that just as at home she would not want anyone to come and see that it is dirty so she feels the same in the hospital. Another cleaner also told us that she cleaned the maternity ward, “any time it looks dirty”.

**Fig 4:**
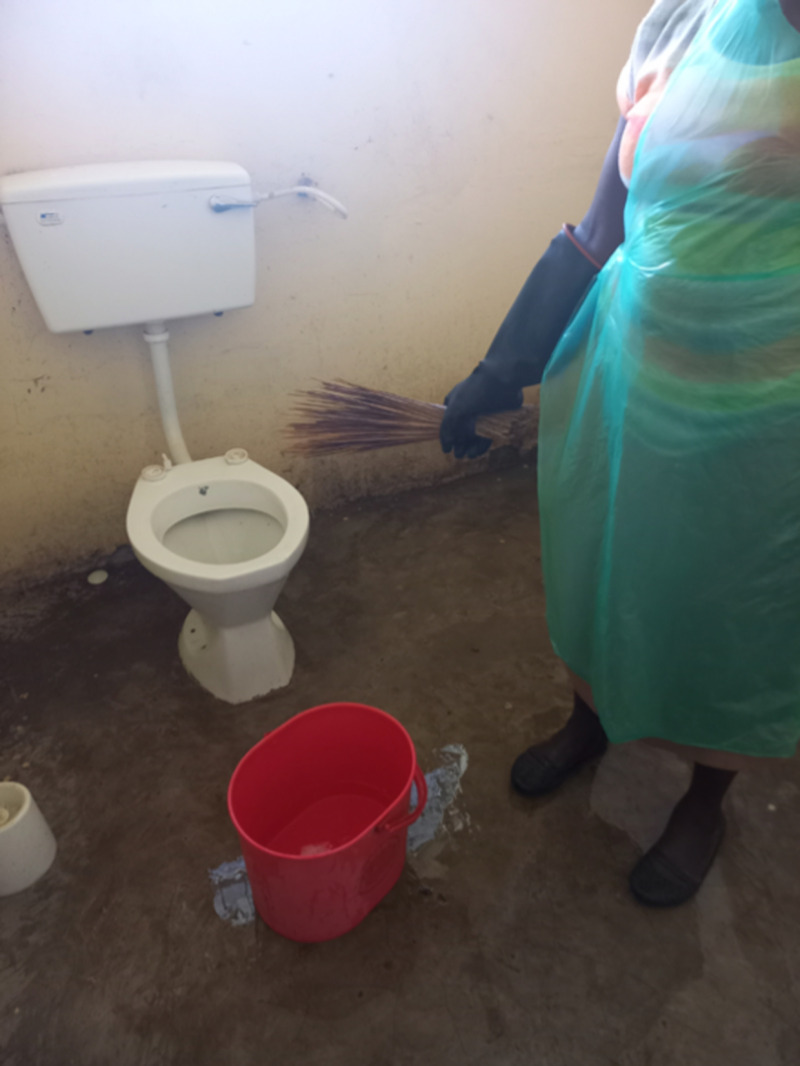
A cleaner poses with the equipment she uses for cleaning (Photo credit: R.Ouda).

Cleaners often lacked equipment that would enable them to do their job safely. All had access to gloves and some had gumboots. One complained to us about that, “she feels that having only one broom for cleaning the rooms, toilet and kitchen is not ideal”, whilst another complained about the very short broom she had been given to clean the maternity toilet (Fig 4). It seemed to us that many of these cleaners were unaware of the risk that they were under. For example, one woman complained that she didn’t have proper PPE for cleaning such as an apron or boots, but rather than risk of infection, her concerns were that this made her feel embarrassed, because sometimes she was forced to walk around the health facility in dirty clothes, and she didn’t like people seeing her that way.

**Fig 5:**
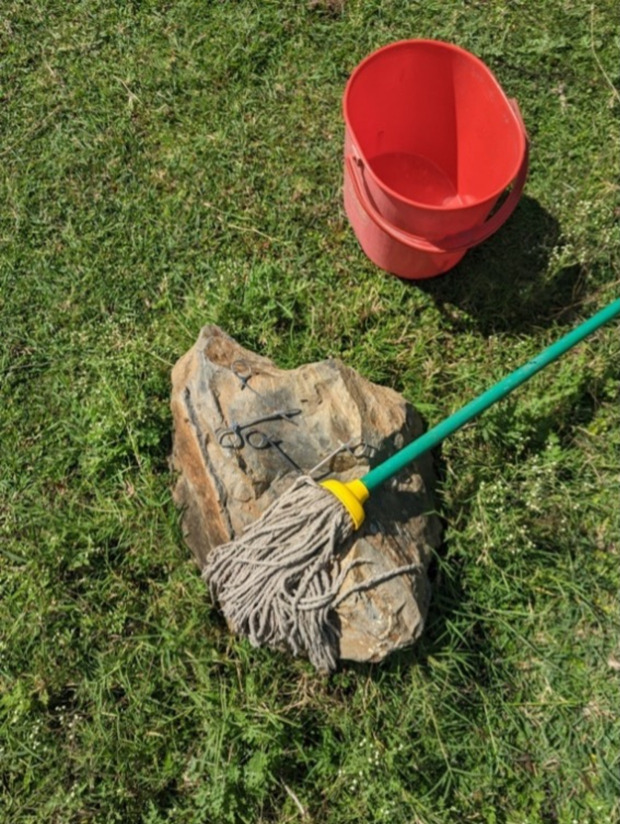
A mop drying on a stone next to medical equipment (Photo credit: H. Brown).

We also observed a number of cleaning practices that were downright dangerous. One cleaner mopped the floor then wrang out water from the mop using her (gloved) hands. Another entered the maternity ward and picked up some sheets that were stained with blood to take them to the laundry, carrying them in her bare hands. In another case we found a cleaner who had left medical equipment and a floor mop both drying together on a stone (Fig 5)

We found that the cleaning staff mostly had access to bleach. Two facilities benefited from machines that produced bleach for their own facility use. However, in one case we observed a cleaner washing a toilet in a maternity ward without soap or bleach, and another complained that they did sometimes run out of bleach. In one facility, stained bed sheets were being soaked in cold water without bleach, then washed by hand.

### Waste

Waste disposal was an issue across all the facilities where we worked. In the whole region, none of the facilities had an incinerator for burning sharps. The largest facility had a burning chamber and the smaller facilities used pits for burning rubbish. This meant that facilities had to store sharps until the county government could organise a collection for incineration at a central place (mostly at the county referral hospitals), which often took months.

All facilities practiced waste segregation as recommended. However, all the facilities complained about the erratic supply of coloured bin liners that were used to colour code waste. Sometimes facilities were forced to use the wrong colour bags in bins and in other cases staff put waste directly into bins without using a bin liner, meaning cleaners would later have to remove the waste and then clean out the bins. Some bins had pedals that allowed people to put waste in the bins without touching them, but not all bins were equipped like this. One nurse-in-charge blamed this situation on a new system of health financing. Previously the health ministry had purchased basic commodities for health centres, he explained, “and then we never used to lack things like gloves, bin liners, or *jik* [bleach]. Now we are supposed to collect user fees and use the money to buy supplies, but what we receive in terms of user fees is not sufficient.” (Facility in-charge, December 8^th^ 2022). On two occasions we were told that placentas were sometimes disposed of in bins without liners, and in one instance that casuals were forced to empty these bins and clean them without wearing gloves. All the four facilities also reported having an IPC committee in place, with most committees having been re-activated within the preceding two years during the COVID-19 pandemic.

### Hand cleanliness

We collected data from a total of 111 adult participants comprising 48 patients (43.2%), 32 visitors (28.8%) and 31 HCWs (27.9%). Participants were mostly female (88 or 79.3%) and the highest percentage interviewed reported last washing their hands within the last 4 hours (70 or 63.1%). For responses to the knowledge question, the majority (82 or 73.9%) respondents most frequently selected the correct response (‘hand washing with soap and water when hands are visibly dirty’). For hand swabs (qPHAT scores), the median score was 5 (on a scale of 0–10) and an interquartile range of 4–6 among all participants and 5 (2–6) among HCWs, 5 (4–6) among patients and 5 (3–6) among visitors.

## Discussion and conclusion

At the time of our research in Dec 2022, the Covid-19 pandemic was a major concern, although the most serious phases of the pandemic had already passed. Across the border in Uganda, there was an ongoing Ebola Virus Disease outbreak, with the nearest case just a few hundred kilometres away from the study site. A US Centres for Disease Prevention and Control (CDC) and Washington State University funded hand hygiene project involving provision of hand hygiene stations, ABHR, liquid soap, training and follow-up observations had been implemented in these facilities since 2021 and was recently completed at the time of this fieldwork [3]. These initiatives and projects were marked by increased procurement of IPC equipment and supplies, training, mentorship, roll out of guidelines and policies. Under the circumstances, we hypothesised that the majority of IPC processes and components would be available and working, and that the health workers would be engaged in heightened observation of IPC protocols.

This was not what we found. Half of the facilities had no HH policy on display, despite these being provided during COVID-19 response. Few health workers or patients were wearing masks and concerns about Covid-19 had almost completely subsided. Except around toilets, hand hygiene resources were largely available, having been supplied during COVID-19 pandemic, and were sometimes placed in close proximity to health care workers, but they were rarely used at the proper moments. In some instances, HH facilities were not placed at strategic locations, despite this being a key area that had been addressed in recent HH projects. Patients and other visitors rarely washed their hands after entering health facilities or after using latrines, and qualified medical staff also frequently failed to observe the WHO mandated points of hand hygiene, for example before and after touching patients. ABHR was available, although not always placed strategically within accessible areas for HCWs. ABHR stock-outs were also common. Some objects in common use were touched by many different people, who were often also touching patients, especially in reception and triage areas.

Other than bin liners and other support for waste disposal, which were frequently deficient, most health facilities had access to sufficient supplies of clean water and basic PPE including gloves, soap, and hand sanitiser. However, previous experience of stock outs meant health workers were making private risk assessments, and saving gloves for those encounters with patients they understood as being most risky. A major finding was that many IPC activities, particularly cleaning, were left to unqualified and untrained support staff, and these mostly female staff were at unacceptably high occupational risk. The evidence we present in this paper also suggests that many improvements in the study setting that occurred during the Covid-19 pandemic were not institutionalized and were already falling away by late 2022 as the perceived risk of Covid infection subsided.

Our results are sobering. They suggest that health workers, patients and wider communities may be placed at considerable unnecessary risk due to poor IPC practice in public hospitals in this region. We observed lack of training and support for cleaners tasked with some of the most risky practices in health care settings, patchy provision of basic supplies, improvised practices of risk management that are a logical response to years or working in health settings without adequate and reliable resources, and a lack of awareness of the considerable personal risk faced by staff in health facilities. The qPHAT scores also show no difference in hand cleanliness between health staff and patients, perhaps pointing generally to low but comparable rates of hand hygiene levels among health staff. If there were another serious epidemic in this region, our research suggests that these health facilities would be ill-placed to respond in ways which would minimise hospital-based transmission of infections. We assume that poor IPC practice in these hospitals may drive numerous preventable infections. Our results help to illuminate the multidimensional nature of IPC gaps in these facilities, and the various ways in which gaps in IPC materialize, whether through failure to adopt or roll out IPC guidelines, poor penetration of IPC strategies or resources in the health system, or instances when implementation is initiated but not sustained.

Most importantly, our research shows that improving guidelines and resolving issues of supply will not be sufficient to create improved IPC in Kenyan health facilities. The facilities that we studied had all recently participated in a number of different projects to improve access to IPC materials and their use. However, IPC practices are socially and culturally determined, and require attention to the multilevel contextual factors that influence people’s behaviours within health settings. There is an urgent need to develop interventions that are sensitive to political, cultural and social-economic context and respond to existing policies and IPC guidelines in ways that allow them to become implemented, whilst maintaining awareness of factors such as the impact of professional biography and previous experience, perceptions of service delivery, community relations, and wider culture, values and preferences [18,19].

## Limitations

This was a small pilot study, involving only four health facilities. Although there is some overlap with findings of similar studies from elsewhere in Africa, our results must be read as suggestive, rather than representative. Throughout the study, we paid particular attention to the impacts of observer presence on behaviours. The nature of ethnographic engagement meant that we were able to record instances – and there were a number of these – where we suspected people were changing their behaviour to ‘please’ us. Paying attention to these moments allowed us to infer inverse behaviours. We also found, as is common in ethnographic fieldwork, that people soon relaxed, reverting to what we assumed we could read as more ‘natural’ behaviour. This also meant that in later conversations, we could also return to earlier moments and discuss how or why people had behaved in certain ways. As a mixed team of Kenyan and European researchers we were able to mitigate some cultural limitations in data interpretation.

## Conclusion and recommendations

Taken together, these results present a disconcerting outlook on the sustainability of capacities cultivated in response to the COVID-19 pandemic. Several authors have previously emphasized the advantages of investments made during the pandemic, contending that they could be exploited to enhance patient care, particularly in infection prevention and control (IPC) [e.g., 3,20,21]. This assertion appears to be unsubstantiated in the context of our study. It is plausible that the lack of sustainability plans by government entities or the absence of efforts to integrate these investments into regular procedures has led to the neglect and deterioration of a significant portion of these investments.

Like other studies, ours shows widespread issues with IPC compliance, particularly handwashing [22], suggesting that IPC interventions in Africa could benefit health institutions in many ways. [23] The key insights of this study, which are made visible by combining our ethnographic and epidemiological findings, relate firstly to the distribution of risk in hospital settings and the way that cleaning staff are disproportionally affected by inadequate IPC regimes, and secondly to the ways that health care workers employ forms of practical wisdom as ways to keep themselves safe, institutionalising behaviours that do not meet WHO guidelines for patient care. Health facilities need to develop clear guidelines for cleaning staff and ensure that they are properly trained and provided with adequate cleaning materials. The more complex task of changing health care worker behaviour will require not only education, given our research shows almost universal *awareness* of WHO guidelines, but interventions that are sensitive to local logics and practical workarounds that health care workers have developed in the face of persistent shortages of basic materials. Prioritising regular and sufficient supplies of IPC materials will no doubt be a key part of delivering sustained behaviour change, as others have also shown [23]. Public health leadership should also prioritize the development of thorough sustainability plans for capacities established during emergency responses, such as the COVID-19 pandemic. These plans should extend beyond the immediate crisis and include strategies for integrating investments into routine healthcare processes. By foreseeing the long-term implications and ensuring proactive measures, leaders can enhance the chances of maintaining and leveraging the benefits of pandemic-driven investments in healthcare infrastructure. Public health leaders should also enforce and monitor the integration of pandemic-related investments into routine healthcare processes. This involves not only making initial investments but also ensuring that resources are effectively utilized in everyday healthcare operations. Including behavioural and social science insights about *why* and *how* practices are integrated into everyday working routines, and what barriers exist to making these changes, is likely to be an important part of these developments.

## Supporting information

S1 Checklist Inclusivity in global research. (DOCX)
